# P-468. Prevalence of Meningococcal Carriage by Serogroup in Infants and Young Children: a Global Systematic Literature Review

**DOI:** 10.1093/ofid/ofaf695.683

**Published:** 2026-01-11

**Authors:** Zeki Kocaata, Lucian Gaianu, Ifeanyi Ubamadu, Laura Taddei, Thatiana Pinto, Hiral Shah, Pavo Marijic, Patrice Carter, Matthew Turner, Andrew Easton

**Affiliations:** GSK, Wavre, Brabant Wallon, Belgium; GSK, Wavre, Brabant Wallon, Belgium; GSK, Wavre, Brabant Wallon, Belgium; GSK, Wavre, Brabant Wallon, Belgium; GSK, Wavre, Brabant Wallon, Belgium; GSK, Wavre, Brabant Wallon, Belgium; GSK, Wavre, Brabant Wallon, Belgium; Health Economics & Outcomes Research Ltd, Cardiff, Wales, United Kingdom; Health Economics & Outcomes Research Ltd, Cardiff, Wales, United Kingdom; Health Economics & Outcomes Research Ltd, Cardiff, Wales, United Kingdom

## Abstract

**Background:**

Invasive meningococcal disease (IMD) can lead to severe complications or death in infants and young children. *Neisseria meningitidis* serogroups A, B, C, W, X, and Y cause most disease. Understanding which serogroups are carried in healthy children, their prevalence and risk factors, and how this varies by region is essential for effective vaccination strategies. The aim of this global systematic literature review (SLR) was to assess meningococcal carriage prevalence (by serogroup, age, and region) and factors associated with carriage, to better inform public health responses and vaccination strategies.

**Figure:**
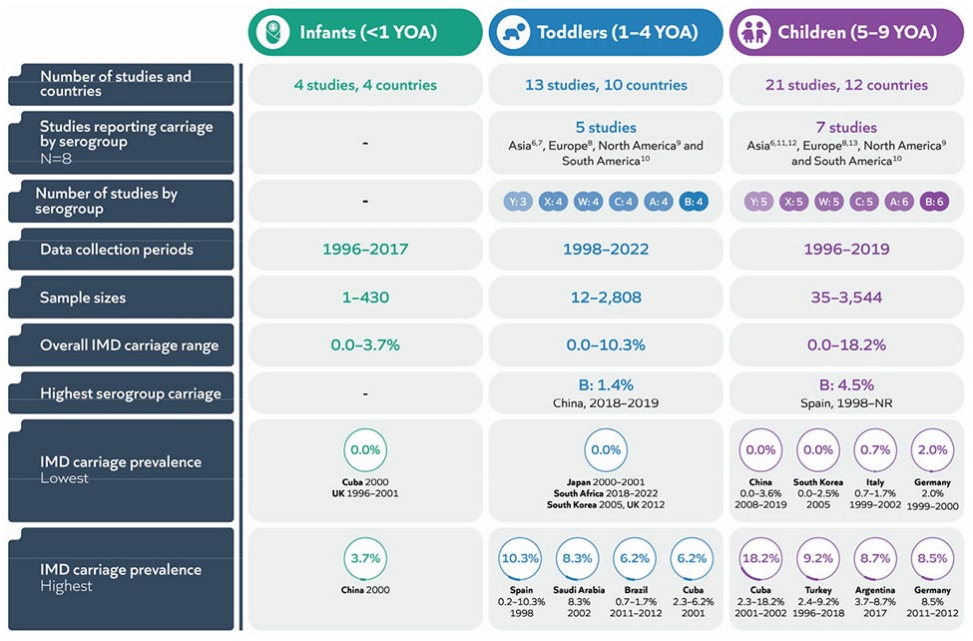
Summary of results IMD: Invasive meningococcal disease; NR: not reported; UK: United Kingdom; YOA: years of age

**Methods:**

The SLR (including Medline, Embase, public health websites) on age- and serogroup-specific carriage prevalence studies (2000 to April 2024, all languages) used predefined selection criteria, adhering to PRISMA and Cochrane guidelines. Studies from the African meningitis belt and on household/case contacts were excluded. Results are presented for infants < 1 year of age (YOA), toddlers 1–4 YOA and children 5–9 YOA.

**Results:**

Of the 203 studies included, 27 reported carriage prevalence in ages 0–9 years, with varying sample sizes and timeframes. Studies most frequently reported serogroup B in toddlers, and serogroups B and C in children. There was wide variation in reported carriage prevalence across countries. Overall prevalence ranged from 0.0–3.7% in infants, 0.0–10.3% in toddlers, and 0.0–18.2% in children. Serogroup B had the highest prevalence: 1.4% in toddlers and 4.5% in children (Figure 1).

Factors associated with carriage were poorly reported and included lower socioeconomic status (SES), body mass index, and pneumococcal carriage in toddlers; and SES, large family size and male sex, pneumococcal carriage, and prior antibiotic treatment in children.

**Conclusion:**

Although IMD prevalence is highest in infants, evidence on meningococcal carriage in infants was limited. SES appeared to be associated with carriage. Serogroup-specific carriage prevalence was highest for serogroup B in this population. Further research is needed to understand serogroup-specific carriage and transmission dynamics to determine vaccination policies in these age groups.

**Disclosures:**

Zeki Kocaata, PhD, GSK: Employee|GSK: Stocks/Bonds (Public Company) Lucian Gaianu, MSc, GSK: Employee Ifeanyi Ubamadu, MSc, GSK: Employee|GSK: Stocks/Bonds (Private Company) Laura Taddei, M.Sc, GSK: employee|GSK: Stocks/Bonds (Private Company) Thatiana Pinto, PhD, GSK: employee|GSK: Stocks/Bonds (Public Company) Hiral Shah, PhD, GSK: Employee|GSK: Stocks/Bonds (Public Company) Pavo Marijic, PhD, GSK: employee|GSK: Stocks/Bonds (Public Company) Patrice Carter, PhD, GSK: Grant/Research Support Matthew Turner, PhD, GSK: Grant/Research Support Andrew Easton, MSc, GSK: Grant/Research Support

